# Improving the clinical impact of biomaterials in cancer immunotherapy

**DOI:** 10.18632/oncotarget.7304

**Published:** 2016-02-10

**Authors:** Joshua M. Gammon, Neil M. Dold, Christopher M. Jewell

**Affiliations:** ^1^ Fischell Department of Bioengineering, University of Maryland, College Park, MD, USA; ^2^ Department of Microbiology and Immunology, University of Maryland Medical School, Baltimore, MD, USA; ^3^ Marlene and Stewart Greenebaum Cancer Center, Baltimore, MD, USA

**Keywords:** cancer immunotherapy, nanotechnology, biomaterials, cancer vaccine, translational medicine, Immunology and Microbiology Section, Immune response, Immunity

## Abstract

Immunotherapies for cancer have progressed enormously over the past few decades, and hold great promise for the future. The successes of these therapies, with some patients showing durable and complete remission, demonstrate the power of harnessing the immune system to eradicate tumors. However, the effectiveness of current immunotherapies is limited by hurdles ranging from immunosuppressive strategies employed by tumors, to inadequate specificity of existing therapies, to heterogeneity of disease. Further, the vast majority of approved immunotherapies employ systemic delivery of immunomodulators or cells that make addressing some of these challenges more difficult. Natural and synthetic biomaterials–such as biocompatible polymers, self-assembled lipid particles, and implantable biodegradable devices–offer unique potential to address these hurdles by harnessing the benefits of therapeutic targeting, tissue engineering, co-delivery, controlled release, and sensing. However, despite the enormous investment in new materials and nanotechnology, translation of these ideas to the clinic is still an uncommon outcome. Here we review the major challenges facing immunotherapies and discuss how the newest biomaterials and nanotechnologies could help overcome these challenges to create new clinical options for patients.

## INTRODUCTION

Over the past 50 years cancer diagnosis, prognosis, and treatment have progressed immensely, improving the outcomes and quality of life for millions of patients each year. Many persistent challenges, however, continue to limit our ability to stop cancer upon detection and to prevent relapse following successful treatment. The barriers undermining these broad goals are diverse, spanning drug toxicity and poor selectivity [1], the immunosuppressive nature of the tumor microenvironment [2], and the heterogeneity of disease across cancers and patient populations [3]. One of the most promising means to address these problems is immunotherapy. This field offers the possibility of treatments that are potent, specific, systemic, and durable–major challenges for invasive surgical resection, and non-specific chemotherapeutics or radiation that frequently result in relapse. In the past decade, exciting progress in immunotherapy–for example, with adoptive therapy [4] and monoclonal antibodies [5]–has stimulated new ideas for harnessing the immune system to combat cancer, while rejuvenating interest in other strategies such as cancer vaccination [3, 6, 7]. However, despite increasing investment in biomedical research, the efficiency with which new treatments are delivered is stagnant [8]. For example, since 1965 the rate of new molecular entities (NME) and new drug approvals (NDAs) by the FDA has remained essentially constant, while the publishing of biomedical manuscripts has increased more than 500% [8]. In cancer immunotherapy, some of the challenges facing new progress include generating immune cells with the correct specificity and function to both recognize and attack cancer cells, maintaining the function of these populations over time, and designing combination therapies that are synergistic without compromising safety [3]. Multidisciplinary approaches focused on clinical challenges in many biomedical research fields, including cancer immunotherapy, could be improved in terms of clinical impact and translatability by integrating capabilities provided by new technology and engineering. In this review we use recent clinical successes and failures to highlight opportunities to leverage nanotechnology and biomaterials to push cancer immunotherapy forward (Table 1).

**Table 1:** Recent studies using biomaterials to address clinical challenges

Clinical challenge/opportunity	Biomaterial strategies	Key references
Toxicity	Localizing and extending release of immunostimulants in materials at safer doses	Kwong 2013^29^, de Titta 2013^32^
Polarizing adoptively transferred T cells	*Ex vivo* T cell expansion with artificial antigen presenting cells	Perica 2015^43^
	Nanoparticle conjugation to T cells	Stephan 2010^45^, 2012^46^
	Implanting T cell seeded scaffolds	Stephan 2015^51^
Polarizing T cells: cell free therapies	Pathogen mimicking microparticles	Pradhan 2014^52^, Singh 2011^53^
Targeting specific pathways	Delivering siRNA with polymer carriers	Alshamsan 2010^65^, 2011^64^; Wang 2013^31^
	Using physiological phenomena to localize therapy in lymph nodes	Liu 2014^39^; Hanson 2015^34^
Revisiting cancer vaccination	Active targeting of nanoparticle vaccines to DCs	Rosalia 2015^102^
	Localized delivery of immune signals with Injectable scaffolds	Ali 2009^109^; Bencherif 2015^110^
	Combining Nanoparticle vaccines with siRNA knockdown of immunosuppressive cytokines	Xu 2014^117^
Combination immunotherapies	Controlled combinatorial delivery of adjuvants	Goldinger 2012^120^, Thomas 2014^121^, Roy 2013^131^, Marrache 2012^135^
Increasing homing and activity of immune cells in tumor microenvironment	Nanoparticle conjugation to T cells	Huang 2015^138^
	Sequestering of immunostimulants in tumors	Liu 2011^140^, Intra 2011^38^
	Nanogel co-delivery of IL-2 and TGF-β inhibitor	Park 2012^143^
Addressing tumor heterogeneity	Microparticle or nanoparticle tumor lysate vaccines	Prassad 2010^154^, Gross 2014^155^
	Capture of circulating tumor cells for identification of neoantigens or tumor cell phenotype	Halo 2014^162^, Azarin 2015^137^

### Overview of biomaterials: Classes and attractive properties

Biomaterials are ubiquitous in biomedical research, and have had some notable impacts in cancer therapy over the past few decades. Thus far, most of these advances–at least clinically–have involved improving the solubility, reducing the toxicity, or increasing the half-life of small molecule chemotherapeutics such as doxorubicin. These improvements illustrate a few of the properties that make biomaterials of great interest for cancer immunotherapy. Speaking generally, “biomaterial” is a term that spans natural or synthetic polymers, lipids, metal contrast agents, engineered cells, quantum dots, and a multitude of self-assembled structures. These materials are often used to build implantable scaffolds or devices [9, 10], as sensitive biosensors on functionalized surfaces within microfluidic devices [11, 12], or to formulate nanoparticles (NPs) or microparticles (MPs) that can be delivered or conjugated to cells *ex vivo* or *in vivo* [13]. One classic advantage of biomaterials is the co-delivery of cargo by encapsulating two or more cargos (e.g., small molecule drugs) in a biodegradable polymeric particle. This approach is frequently used to ensure cells or tissues receive each cargo type to work in synergy, or–by synthesizing polymers with an appropriate degradation rate–to achieve a desired sustained release profile. In addition to co-delivery and controlled release, many particle-based strategies are aimed at improved targeting by surface conjugation of antibodies or ligands for receptors expressed on target cells or tissues.

Another important focus of biomaterials has been in protecting biologic cargo from degradation in the presence of enzymes or extreme pH, and to reduce systemic toxicity by allowing drug to be slowly released over time or upon reaching target tissues such as tumors. This has been particularly important in cancer, where increasing the circulation time of drugs through modification with polyethylene glycol or other molecules has led to better tumor targeting; targeting occurs because of the leaky tumor vascular that causes preferential accumulation at tumors through the enhanced-permeability and retention (EPR) effect [14]. In this last area, liposomes, multi-lamellar vesicles, exosomes, and other lipid-based nanostructures have been particularly useful owing to the highly biocompatible nature of this class of biomaterials [15]. A more recent area of interest is also arising: the intrinsic immunogenic properties of some biomaterials. Many studies demonstrate that common polymers such as poly(lactide-co-glycolide) (PLGA) and poly(styrene) activate pro-inflammatory pathways (e.g., inflammasome) [16–18]. These characteristics, with better understanding, could be exploited to design polymers that serve not only as carriers, but also as agents that help polarize immunity. From another perspective, these materials can complicate rationale design of vaccines and immunotherapies because the carrier itself can alter the response to other vaccine components. Several new strategies are exploring self-assembly of proteins or immune signals to co-deliver vaccine and immunotherapy components [19–21]. In one of these approaches, electrostatic assembly is used to assemble antigens and adjuvants without synthetic polymers or other carrier components [21]. These immune polyelectrolyte multilayer (“iPEMs”) structures thus mimic favorable properties of biomaterial carriers (e.g., tunable sizes, co-delivery) while creating a well-controlled platform for assembling multiple immune signals at high densities without the complicating intrinsic immune effects of many polymers [20, 21].

### Disparity between clinical impact and the investment in nanotechnology and biomaterials

Despite the exciting potential of biomaterials, the impact on the clinic has been modest relative to the pre-clinical investment that has been made. For example, by one recent estimate, 3% of the roughly three million paper published in the cancer field are associated with clinical trials [22]. In contrast, cancer papers also involving polymeric materials only connect to clinical trials of some form in 1% of studies. On the other hand, categories such as liposomes and monoclonal antibodies–both much more clinically-established relative to polymeric materials–are ultimately associated with clinical trials in 4% and 8% of cancer papers, respectively [22]. Though this is a single measure, one interpretation is that as more biomaterial-based strategies advance to the clinic, the potential of other material strategies will receive increasing attention. At present, however, there is a significant gap between clinical impact and the investment in basic and pre-clinical nanotechnology research [22–25]. Below we discuss seven areas where challenges or recent progress in the clinic suggests specific opportunities for biomaterials and nanotechnology to make a significant impact. These include i) toxicities associated with current immunotherapies, ii) reprogramming or polarizing anti-tumor T cells *in vivo* or after adoptive cell therapy, iii) targeting more specific immune pathways, iv) cancer vaccination, v) combination immunotherapies, vi) enhanced homing to and activity of immune cells in the tumor microenvironment, and vii) heterogeneity of disease.

## CURRENT CLINICAL CANCER CHALLENGES CREATE OPPORTUINITES TO HARNESS BIOMATERIALS

### Reduce toxicity and enhance pharmacokinetics (PK) or pharmacodynamics (PD)

Similar to chemotherapy and radiation, the potency of cancer immunotherapy can be accompanied by toxicity severe enough to warrant discontinuation. These effects are challenging to address because most of the existing immunotherapies involve large doses (e.g., IL-2 and interferon alpha) delivered systemically to overcome poor PKs or PDs [1]. Thus, many immunotherapies continue to be limited by poor therapeutic targeting, bolus concentrations, and non-specific side effects. The clinical toxicities unique to immunotherapies–including vaccines, cytokines, checkpoint blockades, and cell therapies–have been recently reviewed by Weber *et al.* [1]. One of the hallmarks of immunotherapy-related adverse events is the induction of unrestrained autoimmune responses toward healthy tissue due to overactive effector cells or due to targeting of tumor-associated antigens (TAAs) that are expressed in both tumors and healthy tissues [1, 26].

Biomaterials can be used to encapsulate and protect therapeutic moieties from biodegradation, to passively or actively improve targeting, and to sustain the release of encapsulated cargo. Each of these features can be harnessed to improve PKs or PDs of immunotherapies and to reduce the associated toxicities. Consequently, it is not surprising that the type of biomaterial that has been most successful in clinical cancer therapy is the use of nanoparticles–liposomes, in particular–for therapeutic delivery of toxic chemotherapeutic drugs [27].

In immunotherapy specifically, clinical testing of liposomal or nanoparticle vehicles for altering toxicity, PK, and PD is under-explored, but a number of pre-clinical investigations demonstrate the exciting potential of these ideas [28–31]. Kwong *et al.* formulated two liposomal therapies that overcome typical toxicity-limited dosing. The first uses liposomes to co-deliver anti-CD40 antibody and CpG oligonucleotide–an immune-stimulatory toll-like receptor agonist (TLRa)–to tumors in a subcutaneous melanoma model (B16-F10) [30]. Compared to soluble delivery, the liposome-coupled therapy resulted in preferential retention of the liposomes and their encapsulated cargos in tumors and the draining lymph nodes (LNs). Notably, the circulating levels of both CpG and anti-CD40 antibody in serum were reduced, leading to significant reduction of pro-inflammatory cytokines such as IL-6 and TNF-α. Alanine aminotransferase (ALT) enzyme–a marker for hepatic damage–was also much lower during liposomal therapies compared with soluble therapies. In a second study, two stimulatory agonists–IL-2/Fc fusion protein and anti-CD137 antibody–were anchored on liposomes, and this therapy was tested against soluble anti-CD137 antibody and IL-2/Fc doses during intraperitoneal (*i.p.*) and intratumoral (*i.t.*) injections [29]. Although these liposomal formulations elicited similar proliferation of T cells *in vitro* in comparison to soluble treatments, the liposomal forms abrogated toxic levels of cytokines in serum *in vivo*. Specifically, mice receiving liposome-conjugated therapies *i.t.* exhibited serum cytokine levels (IFN-γ, IL-6, CCL2, and TNF-α) similar to untreated mice without weight loss, whereas mice treated with matched soluble doses had profound increases in serum cytokine levels that caused shivering, >5% weight loss, and some treatment-related fatalities. Both soluble and liposomal formulations were able to induce complete tumor regression of 60-70% of B16-F10 tumors treated *i.t.* in mice; however, only the liposomal formulations allowed for delivery of larger doses that would have elicited systemic toxicities in soluble form because the liposomes forced sequestration of the therapies in tumors and tumor-draining LNs.

**Figure 1:**
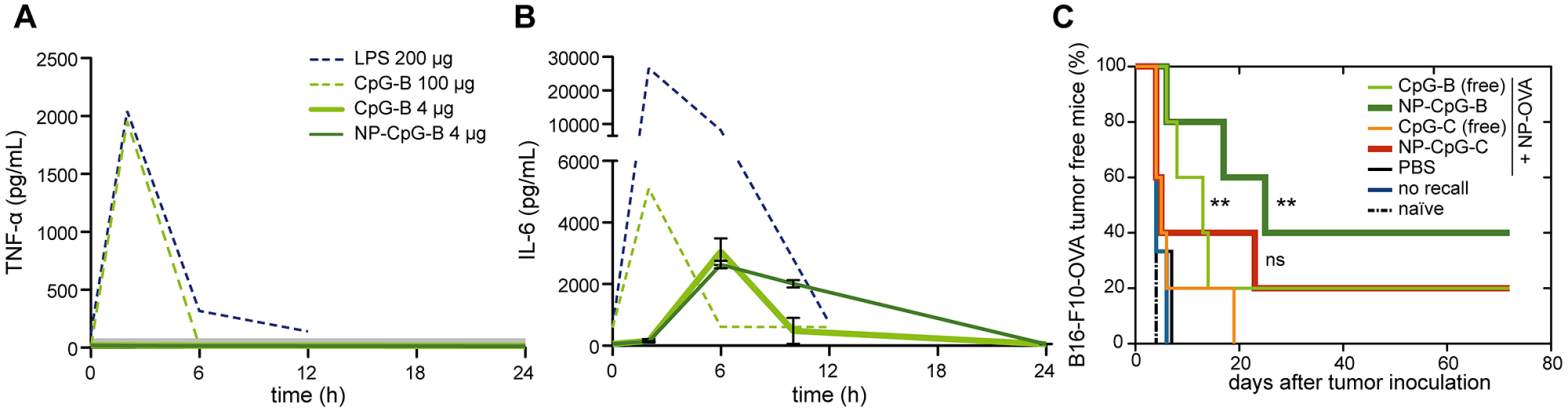
CpG variants formulated in NPs prevent tumor growth better than free CpG without increasing systemic inflammation Low doses of NP-CpG-B do not significantly increase peak levels of inflammation as measured by serum levels of **A.** TNF-α and **B.** IL-6. **C.** NP-CpG-B and NP-CpG-C protects mice from B16-F10-OVA tumor establishment better than unconjugated counterparts. Adapted with permission from [32].

Similarly, several recent investigations have used non-liposomal materials to minimize toxicity by targeting or retaining immunotherapeutic agents inside tissues vital to disease reduction such as tumors and LNs. These approaches have included NPs and MPs [32–35], self-assembling injectable scaffolds [36, 37], and other materials designed to locally deliver, sequester, and/or preferentially trap cargo in tissues of interest [38–40]. In one study, Hubbell, Swartz, and colleagues reported mixed populations of poly(propylene sulfide) NPs conjugated individually to either a model tumor antigen (ovalbumin, OVA) or CpG-based adjuvants. NP conjugation facilitated co-localization of antigen and adjuvant via trafficking of both NP-OVA and NP-CpGs to lymph nodes following intradermal (*i.d.*) injection in mice through size-dependent lymphatic draining patterns [32]. Analysis of systemic cytokine levels showed that the dose of CpG used in both NP and free form was not capable of inducing high systemic cytokine concentrations, indicating a low risk of toxicity (Figure 1A, 1B). In a prophylactic model, low dose NP-CpGs enhanced antigen-specific memory recall of cytotoxic T lymphocytes (CTLs) more than low-dose soluble treatments, resulting in greater CTL expansion and cytokine secretion compared with soluble therapies. Additionally, NP-CpGs delivered alongside NP-OVA conferred complete protection against EG.7-OVA lymphoma and significant protection against B16-F10-OVA melanoma tumors, whereas tumor protection was significantly diminished for B16-F10-OVA tumors when the same low-dose CpG formulations were given as free molecules (Figure 1C). Although this approach was tested in tumor models expressing OVA, these data demonstrate the value of localizing multiple therapeutic components in lymphatic tissues and the power of using a biomaterial delivery system to passively target lymphatics through reduced adjuvant doses that limit toxicity while maintaining efficacy. These preclinical examples also suggest new potential for cancer immunotherapies that have been previously considered infeasible strategies. For example, treatments that clinicians might typically avoid or have previously discontinued due to toxicity might be reexamined and enhanced with biomaterials through encapsulation, controlled release, and targeting. Similarly, as discussed later, therapies like cancer vaccination that have not previously been potent enough for efficacy, may be revitalized through better materials and new nanotechnologies.

### Reprogramming and polarizing T cells

#### Adoptive cell therapy

Although recent years have brought significant clinical successes using adoptive cell therapy (ACT),–an approach in which patient cells are isolated, expanded, and re-infused–some of the continuing challenges include rapid death of transferred cells, poor migration to tumors, and deactivation of cells entering the tumor microenvironment [4, 41]. Current protocols effectively expand and activate T cells *ex vivo*. However, this approach introduces practical challenges that reduce accessibility of treatment owing to the cost and complexity of the protocols. Another challenge facing the field is the limited control over maintaining T cell expansion and polarization after adoptive transfer while minimizing toxicity and off-target effects of modulatory cues (e.g., drugs, cytokines) administered systemically during these treatments.

There are several routes by which arising nanotechnology might help address these challenges: 1) enhancing and simplifying T cell enrichment and expansion procedures, or 2) providing the ability to locally or selectively modulate T cells after transfer. In one recent example, Fadel *et al*. utilized high surface area carbon nanotube-polymer composites to improve *ex vivo* expansion of OT-1 CD8^+^ T cells. Adoptive transfer of these T cells delayed tumor growth in mice bearing B16-OVA melanoma tumors [42]. Another strategy, recently reported by the Schneck group, simplified *ex vivo* T cell isolation and expansion to support ACT using artificial antigen presenting cells (aAPCs) [43]. These aAPCs consist of magnetic iron-dextran NPs conjugated to co-stimulatory CD28-activating antibodies and MHC I molecules bound to TAAs. TAA-specific CD8^+^ T cells are enriched by magnetic isolation after incubation of CD8^+^ peripheral blood mononuclear cells (PBMCs) with aAPCs. The TAA-specific T cells bound to the aAPCs can then be collected and further cultured, while the co-stimulation and antigen presentation provided by the aAPC drives expansion of the cells prior to adoptive transfer to mice. In current methods T cells isolated from PBMCs are cultured with DCs pulsed with peptide tumor antigen after isolation from peripheral blood of patients [44]. Thus the aAPC method may offer a simpler alternative for T cell expansion, eliminating the need for patient specific DCs while also increasing the number of TAA-specific T cells. An additional benefit of the aAPC platform is a reduction in the collection time, where tumor-specific CD8^+^ T cells can be expanded over 1000-fold in as little as one week. The modularity of this design was demonstrated through efficient expansion of T cells specific to a variety of well characterized tumor antigens, including mouse TAAs (Trp2, gp100), human TAAs (NY-ESO1, MART1), and mutated neoantigens from mouse melanoma (B16) or colon carcinoma (CT26). The anti-tumor efficacy of ACT with T cells expanded using this method was tested in mouse models of melanoma. CD8^+^ T cells specific for Trp2 and gp100 were isolated and expanded with aAPCs. Adoptive transfer of these T cells 8 days after inoculation with B16-F10 tumors significantly enhanced survival compared to mice injected with equivalent numbers of aAPC expanded T cells specific for the model antigen, SIINFEKL. Transfer of aAPC-expanded Trp2 and gp100 T cells caused complete tumor rejection in 25% of mice at 30 days after implantation, whereas all mice treated with SIINFEKL T cells reached terminal endpoints by 22 days post challenge. Building on these and other recent approaches, an important next step is to expand cells from patient samples.

Recent work by the Irvine group has demonstrated a powerful pre-clinical strategy to modulate T cell function in mice by synthesizing NPs containing immunomodulatory signals and conjugating these NPs to the membranes of T cells prior to transfer [45–47]. After adoptive transfer, conjugated NPs provide sustained, local interactions with CD8^+^ T cells, allowing autocrine delivery of the drugs from the NPs. The particles in these studies are multi-lamellar vesicles conjugated to T cells through maleimide chemistry. This approach leverages disulfide bonds which form between maleimide functionalized NPs and the naturally occurring excess thiols displayed on T cell membranes. In one report, this platform was used to modify CD8^+^ PMEL T cells with NPs co-encapsulating IL-15 superagonist and IL-21 [45]. T cells from these mice exhibit a transgenic T cell receptor specific for the gp100 tumor epitope. Mice receiving the modified cells exhibited dramatic polarization of anti-tumor T cells toward central memory phenotypes, greatly increasing T cell expansion and persistence compared to transfer coupled with soluble, systemic therapies or unconjugated NPs [45]. The functional impact of this therapy was complete clearance of tumors in all mice bearing metastatic B16-F10 tumors after infusion of NP-conjugated T cells, whereas mice receiving T cells augmented with an identical dose of systemically delivered cytokines all succumbed to the tumors. A subsequent study demonstrated that T-cell-conjugated NPs are sequestered to the immune synapse, providing a direct route to control T cell function or target tumor cells during binding by CD8+ T cells [46]. This potential was demonstrated by conjugating cells with NPs containing inhibitors of Shp1 and Shp2–phosphatases in the immune synapse that downregulate T cell receptor activation [48]–to improve accumulation of T cells in murine prostate tumors and prolong survival of transferred cells. In these studies the NP-conjugated T cells exhibited a 5.7-fold increase in tumor accumulation four days after transfer. Zheng *et al.* recently reported an alternative approach using liposomes targeted to adoptively-transferred T cells *in vivo* through antibody fragments specific for Thy1.1 or IL-2 receptor ligand [47]. This strategy expands on *ex vivo* NP conjugation techniques by allowing NPs to be conjugated to proliferating T cells *in vivo* after transfer.

The studies above highlight some important opportunities to advance ACT by specifically targeting T cells with continued, localized delivery of immunomodulators at low doses. This ability could reduce toxicities and potential autoimmune reactions associated with repeated soluble administration of high doses of immunostimulatory cues (e.g., cytokines, monoclonal antibodies). These properties are increasingly attractive considering the recent successes of a variety of checkpoint blockade therapies targeting CTLA-4 or PD-1 signaling in the immune synapse [49, 50]. Since one current limitation of checkpoint blockade is the effect on bystander T cells [49], local T cell targeting strategies might also offer further opportunities to enhance the specificity of these combination therapies.

**Figure 2:**
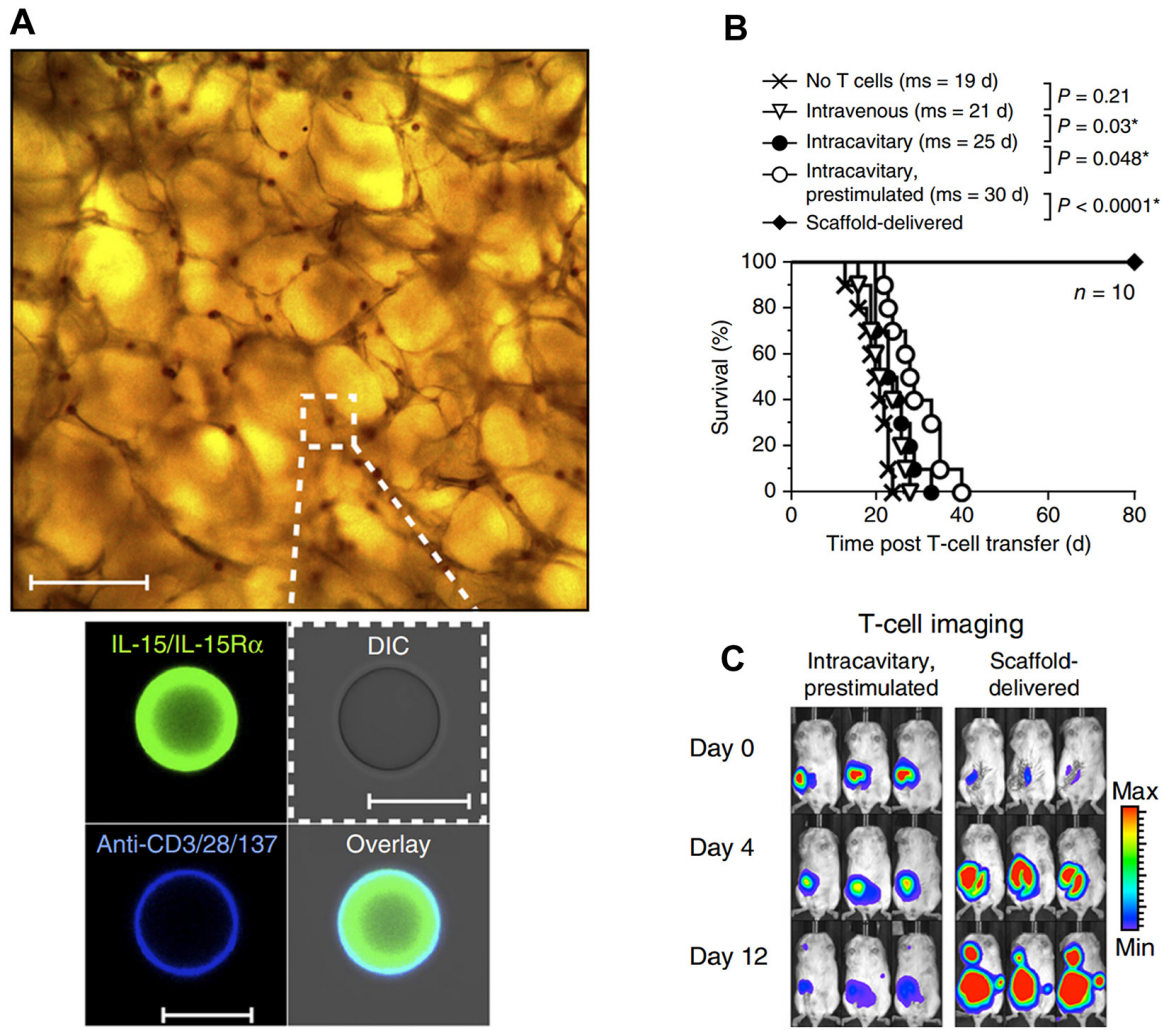
Implantable scaffolds enhance ACT **A.** BMPs encapsulating T cell stimulants are embedded in the implantable scaffold. **B.** Scaffold delivered T cells prevent relapse after tumor resection and greatly enhance survival. **C.** Locally administered T cells within scaffolds persist and proliferate at the tumor site compared to prestimulated T cells administered at the resection site in the absence of a scaffold. Adapted with permission from Macmillan Publishers Ltd: Nature Biotechnology [51] copyright (2015).

An alternative strategy to modifying T cells *ex vivo* is implantation of biomaterial scaffolds seeded with T cells to provide a reservoir that sustains proliferation and activation after implantation. In a recent demonstration of this idea by Stephan *et al.*, T cell-harboring scaffolds encapsulating immune signals were implanted locally near murine breast or ovarian tumors to overcome T cell death, exhaustion, and poor migration to tumors after adoptive transfer–each significant challenges encountered in the clinic [51]. These devices are prepared from alginate scaffolds coated with collagen receptor ligands to support T cell seeding. Additionally, porous silica MPs incorporating IL-15 superagonist and agonistic antibodies for CD28, CD137 and CD3 (Figure 2A) are embedded in the scaffold. These MPs improve T cell proliferation and survival after migration from the scaffold, as demonstrated in an *in vitro* assay measuring the ability of T cells to migrate from the scaffold into 3D tissue mimetic collagen gel loaded with an inflammatory cytokine (IP-10). Implantation of T cell-loaded scaffolds at murine 4T1 breast tumor resection sites greatly enhanced survival (Figure 2B), with all mice treated with the T cell harboring scaffolds surviving and remaining relapse-free throughout the study. In contrast, T cells not loaded in a scaffold but pre-stimulated with IL-15, anti-CD3, anti-CD28 and anti-CD137 only increased the median survival by 5 days relative to untreated mice (Figure 2B). Following local injection at resection sites, these cells persisted transiently (Figure 2C) and became functionally exhausted. T cells injected within scaffolds, in contrast, showed drastically increased proliferation at tumor sites and retained functional, non-exhausted phenotypes characterized by low levels of Tim-3, and 2B4–receptors which inhibit CTL activation and proliferation.

#### Cell free therapies

Immunotherapies aimed at *in vivo* generation of anti-tumor response offer the potential for simpler, more accessible therapies compared with adoptive transfer. However, the efficacy of these approaches has been limited by the need for not just robust anti-tumor response, but control over the phenotypes of the expanded immune cell populations. Biomaterials are being exploited to achieve this goal by offering improved control over the targeting, release, and co-delivery of immunomodulatory molecules compared with soluble systemic dosing. The Roy group recently used polymeric MPs to polarize T cell differentiation *in vivo* by directing the function of DCs [52, 53]. These studies sought to alter the balance between CTL-inducing T_H_1 responses and suppressive T_REG_ or T_H_2 responses by altering the cytokines secreted by DCs during antigen presentation to CD4^+^ T cells. CpG has been largely investigated along these lines during clinical trials due to its ability to induce secretion of IL-12 and other T_H_1-polarizing cytokines. However, CpG also induces IL-10 which can suppress subsequent T_H_1 generations, instead promoting T_H_2 and T_REG_ phenotypes. Pradhan *et al.* thus co-delivered CpG and IL-10 siRNA with plasmid DNA encoding a B cell lymphoma antigen [52]. These signals adsorbed to cationic pathogen mimicking particles (PMPs) built from PLGA and polyethylenimine (PEI) cores. Treatment of DCs with PMPs resulted in an increased ratio of IL-12 to IL-10, a higher frequency of T_H_1 cells, and inhibition of IL-4–a cytokine associated with T_H_2 induction. Implantation of PMPs in a dextran-PEG hydrogel containing MIP-3α–a chemokine which recruits DCs–resulted in enhanced survival in a murine model of B cell lymphoma [53]. Another recent strategy in this area described preferential promotion of a T_H_1 response using chitosan as a carrier for intravesical delivery of IL-12 to promote T_H_1 cytokine profiles, increased survival, and reduced relapse in the MB49 murine bladder cancer model [54, 55]. These results demonstrate the power of using biomaterials to co-deliver immunomodulators for polarizing T cell phenotype to enhance anti-tumor immunity.

As demonstrated by many studies, the CD8^+^ T cell compartment plays a crucial role in eradicating tumors, and in generating long-lived anti-tumor memory populations that can help prevent relapse. CTLs with minimally differentiated phenotypes, including central memory (T_CM_) or T memory stem cells (T_SCM_) demonstrate enhanced proliferative capacity that supports rapid generation of high numbers of anti-tumor effector T cells [56]. However as T cells proliferate, they lose plasticity and differentiate into terminal phenotypes that eventually become functionally exhausted and exhibit reduced anti-tumor activity [57]. Nanotechnology offers the potential to not only generate large populations of anti-tumor CTLs, but to modulate their phenotype to maintain T_CM_ or T_SCM_ to maintain plasticity. For example, recent studies demonstrate sustained release of antigens from particles promotes memory phenotypes compared with the transient antigen presentation characteristic of more traditional vaccines [58]. New strategies that target or sustain the delivery of immunomodulators to regulate differentiation pathways (e.g., mTOR [59]) or induce cytokines that polarize T cells toward T_CM_ (e.g., IL-15, IL-21 [60]) may be a promising strategy to improve current cancer immunotherapies.

### Targeting more specific immune pathways

While clinical immunology is targeting an array of signaling pathways to enhance anti-tumor immunity and suppress tumor-evasion mechanisms, the bulk of biomaterial-based strategies have focused primarily on materials as carriers of established cargos to, for example, sustain delivery. Further, many recent breakthroughs in checkpoint blockade and other areas demonstrate that pathways beyond traditional lymphocyte expansion are important in the efficacy of immunotherapy. Thus, there is substantial opportunity for biomaterials to be harnessed in studying and targeting specific immune pathways. As discussed further in the conclusion, this is a challenging undertaking that requires cross-disciplinary collaboration across engineering and immunology, but these approaches could be transformative.

The potential to harness biomaterials in targeting more sophisticated immune pathways is highlighted by several recent research directions. One pathway of interest is signal transducer and activator of transcription 3 (STAT3), a transcription factor which in addition to regulating tumorigenic and metastatic properties [61], is involved in regulating pro-immune function of tumor cells [62]. In particular for cancer immunotherapy, STAT3 is overexpressed in many tumors and is associated with regulatory pathways that include inhibition of DC maturation and inflammatory cytokine production [63]. Thus, siRNA knockdown to reduce STAT3 expression and the associated tolerance of DCs toward tumor antigens has recently been investigated using PEI modified with stearic acid (StA) lipids to form electrostatic siRNA/PEI-StA complexes [64, 65]. These complexes protect the siRNA from degradation and are readily internalized by tumor cells. When soluble or complexed treatments were administered *i.t.* following *s.c.* inoculation of B16-F10 tumors in mice, the PEI-StA complexes mediated greater anti-tumor effects. Specifically, these particles reduced both the total concentration of STAT3 and the active, phosphorylated form, while also upregulating inflammatory cytokines and classical DC activation markers (CD40, CD86). These effects were also observed with increased tumor infiltration of DCs and CD3^+^ lymphocytes and significantly reduced tumor growth [64, 65]. The non-specific cytotoxicity of siRNA complexes was mitigated in additional experiments by encapsulating the PEI-StA/siRNA complexes in PLGA NPs [65]. Similar efficacy was also achievable using small-molecule STAT3 inhibitors encapsulated in PLGA NPs, demonstrating the robustness of biomaterials to deliver different classes of cargos to target specific pathways [66].

In the examples above, STAT3-inhibitors were administered via *i.t.* injection, which is only feasible in patients with solid tumors accessible for injection. Wang *et al.* recently reported a systemic knockdown therapy using siRNA loaded in liposome-protamine-hyaluronic acid NPs to target CD47, a protein found on the surface of cancer cells that prevents phagocytic destruction [31]. Intravenous administration of these particles in mice every two days beginning on day 8 after B16-F10 tumor inoculation resulted in efficient delivery and retention of siRNA in tumors due to the EPR effect. In these studies, treatment reduced CD47 mRNA five-fold and tumor growth by ∼93% [31].

Beyond targeting cellular pathways co-opted by tumors, pathways that control physiological structures and immune cell trafficking to lymphoid tissues can also be harnessed in new strategies that emerge from immunology and materials science [16]. The benefits of targeting lymph nodes are apparent because lymphatic tissues are key sites where antigen presenting cells and lymphocytes interact to mount anti-tumor responses. One fascinating new strategy to shuttle vaccine cargos to lymph nodes exploits the natural trafficking of albumin from blood, across lymphatics, and into lymph nodes. In these studies by Liu *et al.*, CpG and antigenic peptides were covalently modified with albumin-binding domains to allow “hitchhiking” of these immune signals to lymph nodes [39]. This vaccine method was highly potent and generated efficient immune responses in mice against a variety of antigens, including tumor antigens that caused regression of TC-1 tumors and slowed growth of B16-F10 tumors. Another recent strategy for targeting lymph nodes uses intra-lymph node injection to introduce degradable polymer depots to lymph nodes for local sustained release of adjuvants and other immune signals in these tissues [67, 68]. This approach generates potent antigen-specific responses in mice and could offer a direct route for locally engineering the lymph node microenvironment to control specific immune pathways [67].

**Figure 3:**
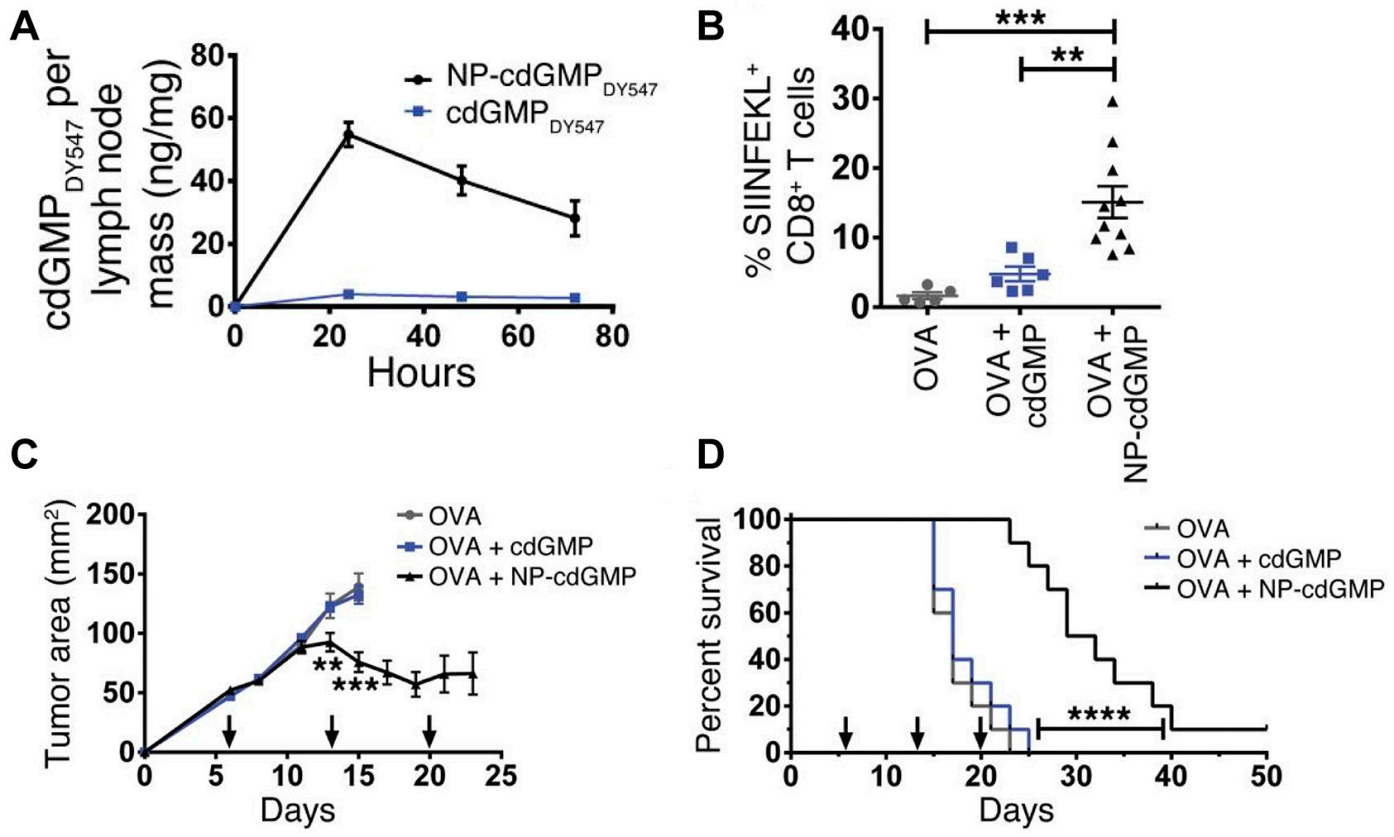
STING agonists exhibit enhanced adjuvant properties and anti-tumor efficacy when administered in NPs rather than soluble form **A.** Only NP-formulated cdGMP accumulates in lymph nodes. **B.** Following *s.c.* inoculation of EG.7-OVA tumors, NP-cdGMP elicits higher antigen-specific T cell frequencies (SIINFEKL) among CD8^+^ cells compared with soluble cdGMP. **C.** Tumor growth is attenuated by NP-cdGMP, but not soluble cdGMP, when administered therapeutically (arrows) after tumor inoculation. **D.** Mice receiving NP-cdGMP exhibit improved survival. Adapted from Figures 1 and 2 of [34] with permission.

Lastly, some very recent strategies simultaneously make use of physiological organization of immune cells and intracellular signaling pathways. Activation of the stimulator of interferon genes (STING) complex, for example, is now known as a major mechanism the innate immune system uses to sense tumor cells [69]. STING agonists like cyclic di-GMP (cdGMP, a bacterial cyclic dinucleotide) are internalized by immune cells after binding STING and are able to activate inflammatory transcription factors such as those that drive type I interferon production. Like many adjuvants, STING agonists may exhibit poor activity in soluble form, partly due to the fact that these molecules do not accumulate in a free form near immune cells in tissues like lymph nodes [67, 70]. In a recent study, subcutaneous administration of soluble cdGMP showed very low accumulation in lymph nodes, whereas nanoparticle formulations (NP-cdGMP) increased cdGMP accumulation ∼15-fold and sustained its presence in lymph nodes using the same injection route (Figure 3A) [34]. To test functionality, mice were inoculated with EG.7-OVA tumors then vaccinated *s.c.* with either OVA or OVA combined with soluble or NP-formulated cdGMP. Vaccination with NP-cdGMP and OVA stimulated the highest frequencies of antigen-specific CTLs (Figure 3B), along with robust antibody production equivalent to the levels achieved with a 30-fold higher soluble dose. Moreover, NP-cdGMP therapeutic vaccination on days 6, 13, and 20 following EG-OVA tumor inoculation attenuated tumor growth (Figure 3C) and prolonged survival (Figure 3D) [34]. Each of the above examples demonstrates the utility of combining detailed immunological knowledge with the features of biomaterials, underscoring the need to cultivate effective cross-disciplinary collaborations and strategies in future studies.

### Renewed potential of cancer vaccination

The success of several new immunotherapy strategies in the clinic have also revitalized the potential of cancer vaccination. One important example is targeting of the immune checkpoints, CTLA-4 and PD-1, which have been highly effective in recent trials [49, 50]. Ipilimumab, an antibody targeting CTLA-4, was the first approved checkpoint blockade therapy, approved in 2010 for treatment of melanoma. In phase III trials conducted on patients with advanced melanoma, Ipilimumab conferred durable and long-term response in a subset of patients, with greater than 20% of patients surviving for over 4 years [50, 71]. Another example is the recent approval of sipuleucel-T (Provenge^®^). Sipuleucel-T is a vaccine consisting of autologous DCs pulsed with a fusion protein of human GM-CSF linked to a prostate cancer antigen (PAP) expressed in more than 90% of prostate tumors [72]. Phase I and II clinical trials demonstrated that sipuleucel-T is safe and can generate PAP-specific immune response [72]. In phase III clinical trials, patients treated with sipuleucel-T had enhanced survival compared to patients treated with a vaccine in which DCs were reinfused without being pulsed with the PAP fusion protein. [72, 73]. These benefits secured FDA approval of sipuleucel-T for treatment of minimally symptomatic or asymptomatic castrate-resistant prostate cancer in 2010, and a variety of clinical trials investigating sipuleucel-T are ongoing [74]. With these recent demonstrations of the renewed potential for therapeutic cancer vaccines, biomaterials are positioned to help overcome the persisting hurdles.

One of the fundamental challenges facing cancer vaccines in the clinic is poor immunogenicity of TAAs. Biomaterials can help combat this challenge by improving the likelihood that vaccine components reach DCs or other immune cells or tissues together. TLRas, for example, are being used to enhance TAA-specific immune response; however, systemic co-administration of soluble TLRas with TAAs does not ensure that both components reach DCs together. Additionally, soluble vaccine adjuvants are rapidly cleared *in vivo*, and may not persist long enough to strongly activate DCs-effects that could limit efficacy, or even promote tolerance against TAAs if DCs present the antigens after adjuvants are cleared [75]. Biomaterials have been utilized to address these limitations by co-delivering and sustaining the release of multiple classes of vaccine cargos (e.g., antigens, ajduvants). Recent approaches along these lines have exploited biomaterials to adsorb, self-assemble, or encapsulate whole tumor cells [76], tumor lysates [77–80], proteins [10, 81–83], minimal epitope peptides [84–89], DNA [90] and mRNA [91].

In addition to increasing immunogenicity of TAAs by providing co-delivery with immunostimulants, biomaterials have also been utilized to facilitate cross-presentation of antigens for the generation of CTL responses [92]. Antigens delivered with particulate carriers can enhance processing of antigen onto the MHC-I pathway compared to soluble delivery of exogenous antigen [93–95]. Additionally pH responsive carriers have been used to enhance this effect by efficiently delivering antigens into the cytosol of APCs. A variety of biomaterial carriers have been developed to release antigens within the acidic pH of endosomes/lysosomes [96–98], and some have been utilized to the promote endosomal escape of released antigens by causing lysis of endosomes [99–101].

Another key advantage of biomaterial vaccine delivery systems, in particular for MPs or NPs, is the ability to preferentially target DCs, which specialize in phagocytosing particulate matter such as cells, antigens, or vaccine particles. The targeting of NPs to DCs can actively be enhanced through the conjugation of antibodies or other specific ligands. Rosalia *et al.* recently demonstrated that NP cancer vaccines can be enhanced by targeting DCs using PLGA NPs conjugated to CD40 antibody [102]. These particles were used to co-encapsulate and deliver the model antigen OVA with Pam3CSK4 (TLR1/2) and Poly IC (TLR3). CD40-NPs effectively targeted and accumulated inside DCs in lymph nodes, driving increased antigen internalization and enhanced expression of DC activation markers. Therapeutic vaccination regimens with CD40-NPs also drove enhanced survival of mice implanted with B16-OVA tumors [102].

Beyond the use of biomaterials as passive vaccine delivery vehicles, a developing area in the biomaterials and vaccine fields is the intrinsic and self-adjuvanting properties of polymers and other materials [16]. Some of the materials now being studied along these lines for cancer vaccination include cationic lipids [82, 83], or polymer micelles [103], dendrimers [87, 88], polyanhydrides [81], self-assembling peptides [84], and fibronectin [86]. These intrinsic immunostimulatory effects–activation of the inflammasome [17], for example–seem to result from the features that many biomaterials share with pathogens: a particular nature/form, repetitive polymeric structures that loosely resemble the repetitive nature of bacterial polysaccharides or other pathogen-associated polymers, and hydrophobic moieties or regions that activate pathogen/danger-associated molecular patterns (PAMPs/DAMPs) [104]. Intriguingly, recent studies by Andorko *et al.* have also revealed that the intrinsic immunogenicity of degradable polymeric carriers can change during degradation [18]. Understanding how the properties of materials exhibit or drive immunostimulatory pathways could improve DC targeting or activation to create a new generation of vehicles that serve not just as carriers but that also help actively direct immune response during cancer vaccination.

Several of the NP-based strategies discussed in previous sections are also relevant to improved cancer vaccination. The aAPCs discussed earlier for *ex vivo* expansion of anti-tumor T cells, for example, have also recently been investigated as vaccines to prime effective anti-tumor CTLs *in vivo* [105, 106]. Additionally, particulate vaccine delivery systems generally accumulate in lymphoid organs through passive drainage, which serves as a passive targeting system for uptake and processing of vaccine particles by lymph node-resident APCs [32]. This process also helps ensure vaccine components encapsulated in particles reach APCs together to maximize the enhancement to antigen processing and presentation, and the subsequent expansion of tumor-specific lymphocytes. As discussed above, there are also some other exciting new strategies for active targeting, including the work of Liu *et al.*, in which albumin shuttling was exploited in mouse models of melanoma and cervical cancer [39].

Whole cancer cell based vaccines have been widely investigated in the clinic and have demonstrated the potential of localized co-delivery of tumor antigens and immunomodulators. These strategies aim to overcome the generally poor immunogenicity of cancer cells by modifying the cells with a variety of immune signals to amplify the response against cancer antigens [107]. A common example of this strategy is whole cancer cells engineered to express the immunostimulant, GM-CSF, which have been studied as a vaccine for a variety of cancers [108]. In these approaches, allogenic or autologous tumor cells are transduced to express high levels of GM-CSF *in vitro* and are then administered to patients. However these strategies have resulted in limited efficacy, likely due to cell death. This hurdle leads to poor persistence and co-localization of the signals and tumor antigens from the engineered cancer cells. To address these limitations, injectable or *in situ* forming scaffolds have been widely studied as next generation cancer vaccines [10, 76–80, 90, 109]. In addition to vaccine delivery vehicles, scaffolds provide sustained delivery of multiple immune signals and can function as sites for robust recruitment, activation and priming of DCs against TAAs *in vivo*. Scaffolds delivering a variety of immune cues have been investigated, including chemokines (CCL20) or cytokines (GM-CSF, Flt3L) for DC recruitment, TLRas (CpG, PolyIC, MPLA) for activation of DCs, and antigens (tumor lysates, model antigens) for the generation of strong and specific anti-tumor response [10, 76–80, 90, 109].

The Mooney Lab is one of the pioneers in this area, developing PLGA or other polymer-based scaffolds to serve as a platform for local juxtaposition of multiple immune signals to promote effective anti-tumor response [10, 76–80, 109]. Work by Ali *et al.* revealed these scaffolds function to enhance anti-tumor immunity by recruiting and priming phenotypically distinct populations of DCs towards TAAs [109]. Scaffolds encapsulating GM-CSF, for example, promote infiltration of conventional DCs (cDCs) that secrete high levels of T_H_1 polarizing IL-12, and incorporation of CpG in the scaffold results in infiltration by plasmacytoid DCs (pDCs) that secrete high levels of type-1 IFNs. The recruitment of heterogeneous populations of DCs corresponded with high amounts of CTLs specific for Trp2–a melanoma antigen–infiltrating tumors, and increased ratios of CD8^+^ effector T cells to T_REGs_ in tumors. These effects provided therapeutic anti-tumor immunity in the B16-F10 mouse model, where implantation of vaccine scaffolds 9 days after the establishment of tumors resulted in survival of 47% of mice at the end of the 100 day study, while all mice vaccinated with GM-CSF-secreting irradiated B16-F10 cells succumbed by 36. Bencherif *et al.* recently demonstrated this strategy can be expanded to scaffolds consisting of implantable cryogels–porous hydrogels formulated at subzero temperatures to mimic tissue properties–for co-delivering irradiated whole B16-F10 cells, CpG and GM-CSF. These cryogel-based scaffolds can be injected with a syringe [110], demonstrating a minimally invasive alternative to surgically implantable scaffolds while still conferring similar therapeutic anti-tumor immunity. Vaccination with the cryogel scaffold vaccine 3 days after tumor challenge resulted in the survival of over 40% of mice during the 100 day experiment, compared to mice vaccinated with bolus vaccine which all reached terminal end points by day 38 post-challenge [76].

The clinical success of immunotherapies targeting immune checkpoints has sparked interest in combining vaccination with these ground breaking therapies. Through this combination, pathways that restrain pro-immune function might be released to create opportunities for vaccines to generate efficacious responses. For example, GVAX, a whole cell pancreatic cancer vaccine utilizing GM-CSF-expressing tumor cells showed initial promise but was ultimately halted in phase III clinical trials due to lack of efficacy [111]. GVAX is now being investigated in combination with PD-1 blockade (Nivolumab) during an ongoing phase II clinical trial [112, 113]. Additionally, sipuleucel-T is recruiting for phase II clinical trials investigating combination with CTLA-4 blockade ipilumab [114].

The idea that GVAX vaccine may be enhanced by the addition of checkpoint blockade therapies highlights one important area related to this idea of combination therapy–how to overcome the immunosuppressive microenvironment of tumors. Although vaccines may generate anti-tumor immune responses, these cells are often inactivated after infiltrating tumors. In addition to the expression of checkpoint blockade ligands by tumor cells, the anti-tumor efficacy of infiltrating immune cells may also be inhibited by suppressive cytokines such as TGF-β [115] or enzymes such as indolamine 2,3-dioxygenase (IDO) [116] secreted by tumor cells or tumor-resident suppressive immune cells (e.g T_REG_, myeloid derived suppressor cells (MDSC)). These immunosuppressive signals can be targeted using small molecule inhibitors or siRNA as a strategy to overcome tumor immunsuppression. However, the effectiveness of systemic delivery of these cargos is limited by poor targeting to tumors and rapid degradation.

Biomaterials have recently been used to improve the delivery of immunomodulators targeting a variety of immunosuppressive pathways to actively enhance the cancer vaccination. Xu *et al.* recently demonstrated the potential to combine biomaterial based immunotherapies and vaccines to alter the tumor microenvironment for enhanced immunity in the B16-F10 mouse melanoma model [117]. In this study, lipid-calcium-phosphate NP (LCP NPs) based vaccines co-encapsulating Trp2 and CpG were first administered subcutaneously to generate a robust anti-tumor CTL response. This NP vaccine was augmented by intravenous injection of TGF-β siRNA delivered in liposome-protamine-hyaluronic acid NPs (LHA NPs)–a formulation the investigators optimized for cargo protection and efficient delivery to the tumor site [117]. LHA NPs efficiently knocked down up to 50% of TGF-β expression in tumors, while leaving TGF-β expression in the spleen and lymph nodes unaffected. The effect on tumor growth was investigated using both early (4 days post tumor challenge) and late stage (13 days post tumor challenge) therapeutic vaccination regimens. The early vaccine regimen was sufficient to clear small established tumors, while late stage vaccination, representing advanced disease with highly suppressive tumors, was ineffective. Systemic CTL responses generated by both vaccines was similar. This finding was demonstrated using an *in vivo* cytotoxicity assay which tested the ability of vaccinated mice to lyse splenocytes from naïve mice, after the splenocytes were pulsed with Trp2 peptide and adoptively transferred. However, the late stage vaccine was able to match the anti-tumor efficacy of early vaccination only with the combination of LCP NP-mediated TGF-β knock down. Importantly, mechanistic investigations revealed TGF-β knockdown depleted T_REGs_ in tumors and caused higher levels of CD8^+^ T cells infiltrating tumors in late-stage vaccination. These effects were minimal following immunization with the monotherapy vaccine. Together, the ideas and examples discussed in this section demonstrate the ability of biomaterials to enhance pre-clinical cancer vaccination, but these combination approaches still remain uncommon and are largely untapped in the clinic, highlighting an important investment area that could “rescue” or improve previously ineffective strategies such as cancer vaccination.

### Combination therapies

With recent developments in immunotherapies, new attention is warranted to strategies combining immunotherapy with not just checkpoint blockade, but also with chemotherapy, radiation, ablation, or resection. This year the FDA granted accelerated approval for the first combination of cancer immunotherapies–checkpoint inhibitors nivolumab and ipilimumab–following phase II studies in patients with unresectable stage III or IV BRAF V600 wild-type melanoma [118]. However, the potential toxicity and difficulty in delivering multiple cargos to tumors or other target tissues is amplified in combination therapies [118]. Material delivery systems may help address this challenge by improving control over existing combination therapy schemes and make new multi-pronged therapies possible through controlled release, dose-sparing, or better targeting during radiation and ablation.

One promising area already being explored as a combination therapy using biomaterials is the stimulation of multiple TLRs through co-delivery of multiple TLR agonists (TLRas) [119–121]. Signaling through specific TLRs occurs because TLRas are recognized by receptors for their pathogen associated molecular patterns uncommon in mammals, such as double stranded RNA (e.g., polyIC, TLR3a) or endotoxins characteristic of certain pathogen categories. These TLRas can be used to drive inflammation and can amplify antigen-specific immune responses. Interestingly, recent work has shown that mixtures of TLRas can be used to elicit synergistic adjuvant responses that are distinct from the responses attributed to the administration of individual TLRas [70, 122–126]. Further, many biomaterial approaches aim to create pathogen-mimicking particles that incorporate multiple stimulatory signals as an alternative to the inactivated or attenuated pathogens often found in vaccines [92, 127, 128]. Virus-like particles (VLPs) are one good example, as these particles mimic viruses through intrinsic immunogenicity resulting from self-assembly of isolated viral proteins without use of viral genomic material.

**Figure 4:**
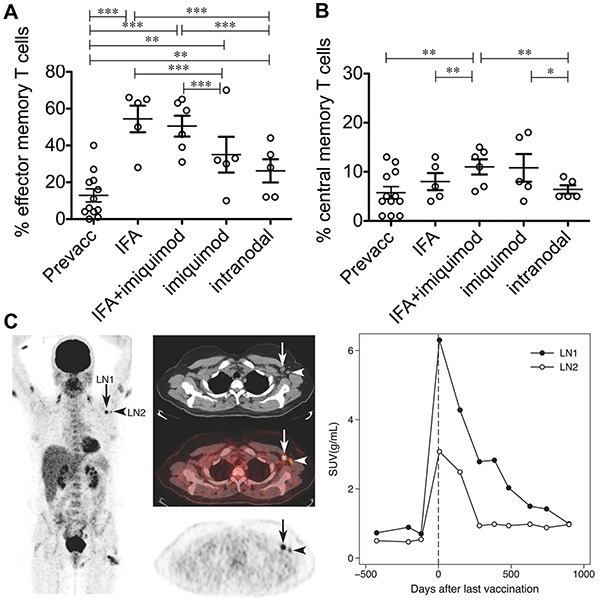
A VLP-peptide vaccine containing CpG delivered s.c. or i.d. with adjuvants, or intra-nodally without adjuvants modulates antigen-specific T cell immunity VLPs with adjuvants promote **A.** higher overall frequencies of effector memory T cells (CCR7^−^/CD45RA^−^), and **B.** higher frequencies of central memory T cells (CCR7^+^/CD45RA^−^) among antigen-specific T cells. **C.** PET/CT imaging of a draining lymph node in a patient receiving subcutaneous treatment (IFA only). Shown is enlargement (arrows), inflammation visible both arms and one thigh (vaccination sites), and sustained increases in glucose metabolism after vaccination indicated by standard uptake value (SUV) measurements. Adapted by permission from Macmillan Publishers Ltd: European Journal of Immunology [120] Copyright Wiley-VCH Verlag GmbH & Co. KGaA. (2012).

VLPs known to accumulate in draining lymph nodes were recently studied alongside TLRas in a phase IIa human trial with stage III and IV melanoma patients [120, 129]. The VLPs were derived from a bacteriophage then loaded with TLR9 agonist and a peptide derived from the Melan-A/MART-1 melanoma tumor antigen. The VLPs were administered in a multi-injection regimen using one of four formulations: i) *s.c.* with Incomplete Freund's Adjuvant (IFA), ii) *s.c.* with IFA and a TLR-7 agonist (Imiquimod) applied topically at the injection site, iii) *i.d.* with topical Imiquimod application, and iv) intra-lymph node injection using ultrasound guidance [120]. Doses were matched for each immunization, except group IV which received lower doses. All patients experienced increased antibody production, with the highest levels in patients receiving IFA. These patients all experienced at least two-fold increases of Melan-A/HLA-A2 tetramer positive T cells in peripheral blood. Vaccines containing IFA also increased frequencies of effector memory T cells most efficiently (Figure 4A), while Imiquimod best expanded antigen-specific central memory T cells (Figure 4B). PET/CT scans from a patient that received *s.c.* immunization revealed swelling in draining lymph nodes, as well as local inflammation at vaccine sites, confirming drainage to lymphatics still plays a role in this vaccine's efficacy (Figure 4C). This particular patient was studied for an extended period and showed increased metabolic response activity in draining lymph node hundreds of days after the last vaccine dose (Figure 4C) [120]. Moreover, because particle size influences particle trafficking and retention, preferential drainage of these VLPs to lymph nodes near vaccine sites may explain some of the inflammation and metabolic activity observed in patients’ lymph nodes using the peripheral injection routes. These outcomes indicate a role for the addition of multiple TLRas administered via distinct routes.

Another new combination therapy being tested in the B16-F10 melanoma model exploits the size of nanoparticles to preferentially deliver CpG and paclitaxel to tumor draining lymph nodes [121]. Here, both CpG and paclitaxel serve as TLR agonists (TLR9 and TLR4, respectively), rather than the more traditional role of paclitaxel as a chemotherapeutic. Particles loaded with both agonists activated DCs *in vitro* with no cytotoxicity. Four to nine days after implanting B16-F10 tumors, the NPs were administered *i.d.* to limbs of mice on the same or opposite side of the tumors as an *in situ* vaccine. This experiment studied immune responses toward tumors directed only by TLR stimulation and the natural levels of tumor antigen expressed in the mice, instead of administration of additional tumor antigen. Particles administered on the same side and loaded with both CpG and paclitaxel accumulated in the tumor draining axillary and brachial lymph nodes, causing the greatest reduction in tumor growth [121]. These studies revealed that both the combination of different TLRas and the accumulation of particles in the tumor draining lymph node each influenced the therapeutic outcomes without inclusion of a tumor antigen. Thus new approaches should leverage biomaterials to break tumor tolerance and promote infiltration of immune cells that discover new tumor antigens for improved adaptive response.

Although in the clinic chemotherapeutics are regularly combined with immunotherapy, application of biomaterials to this area has been relatively sparse [130, 131]. This clinical promise however, along with the ease of co-encapsulating small molecule drugs (e.g. paclitaxel, doxorubicin) and biological molecules (e.g. TLRas), warrants more investment. In one recent example, a derivative of the TLR4 agonist LPS and paclitaxel were encapsulated in PLGA nanoparticles [131]. Peritumoral delivery of the particles elicited stronger anti-tumor effects, increasing the frequencies of tumor-infiltrating DCs (CD11c^+^), macrophages (CD14^+^), and CD4^+^ and CD8^+^ T cells while driving a 40% reduction in tumor burden relative to paclitaxel delivered alone [131]. Further study of these types of combinations may reveal opportunities to build on and optimize material platforms to enhance the individual effects of each therapeutic.

Radiation and ablation also provide unique opportunities for combination with immunotherapy because recruitment of immune cells to the local tissues in the treatment area can allow identification of neoantigens by immune cells infiltrating these sites. This mechanism has been hypothesized as a potential explanation for tumor regression observed in areas of the body not directly treated via radiation or ablation [132–134]. In one effort to exploit this effect, researchers combined a polymeric NP platform with gold NPs, CpG, and photosensitive zinc phthalocyanine [135]. These complex NPs enable photothermal ablation while simultaneously delivering a TLR agonist. This approach might create an *in situ* vaccine by delivering an adjuvant to sites where ablation kills tumor cells and exposes additional tumor antigens. Thus far, however, the platform has been investigated *in vitro*. For these studies, 4T1 tumor cells were ablated with the NPs and exposed to DCs. The DCs were activated by the TLRa-loaded NPs and phagocytosed the remnants of the ablated tumor cells [135]. This strategy represents a rational approach toward combining multiple therapies: ablation and immunotherapy. Building on this broad concept, researchers should explore new approaches for using immunomodulatory agents to create *in situ* vaccines by combining them with treatments like radiation, ablation and surgeries that expose new tumor antigens. Moreover, as we learn more about the unique connections between each arm of combination therapies, this synergy may also be able to help reduce relapse, for example, by promoting memory T cell populations specific for neoantigens.

### Increased homing of immune cells to the tumor microenvironment and improved activity at these sites

Despite the clinical success of some immunotherapies and vaccines in generating tumor specific CTLs, the anti-tumor efficacy of these treatments is limited by poor migration to tumors, as well as inactivation of cells that do reach the tumor microenvironment [2]. Biomaterials offer the potential to specifically target the tumor microenvironment through the EPR effect, as well as exciting new strategies to alter the trafficking of immune cells and cancer cells. This section discusses these advances.

In addition to targeting tumors at the tissue level, NP size can be tuned to passively target specific immune cells within tumors. Kourtis *et al.* demonstrated 30nm poly(propylene sulfide) NPs administered *i.d.* accumulate at high levels in the spleen and draining LNs, and are efficiently taken up by cellular populations of the myeloid lineage. In particular, NPs are efficiently internalized by myeloid derived suppressor cells (MDSC) in tumors, tumor-draining LNs and spleens. This represents a potential clinical strategy to deliver immunomodulatory cues to suppressive cell subsets [136]. Another approach integrating passive targeting of tumor cells–discussed below in the heterogeneity of disease section–was recently developed by the Shea lab to recruit tumor cells to a polymeric scaffold implanted at a distant site from the tumor [137].

NPs may also be actively targeted to lymphatic tumors for the delivery of therapeutics by taking advantage of the ability of lymphocytes to home to lymphoid organs. Huang *et al.* demonstrated a strategy to deliver NPs encapsulating a chemotherapeutic (SN-38) to lymphoma tumors through the conjugation of NPs to polyclonal T cells from C57BL/6J mice [138]. T cells were expanded *ex vivo* in the presence of rapamycin, which polarized T cells toward phenotypes that retain high levels of lymphoid homing receptors (e.g., CD62L, and CCR7) after expansion. NP-conjugated T cells effectively trafficked to lymphoid tumors after adoptive transfer, causing increased concentrations of SN-38 in these lymph nodes compared to administration of unconjugated NPs. This active targeting of tumors resulted in increased survival of tumor bearing mice at greatly reduced doses.

The above examples demonstrate the benefits of passively targeting tumors after administration at a distant site from the tumor. In some cases, *i.t.* injection of non-resectable tumors offers a simple route to directly deliver immunostimulants that locally reactivate exhausted immune cells in the tumor microenvironment. By causing these reactivated APCs to be primed against TAAs within tumors, localized *i.t* therapy may thus offer exciting potential to generate systemic anti-tumor immunity to combat distal tumors, or memory that helps address relapse [139]. Using biomaterials with this strategy capitalizes on the ability of particulate material to be retained in tumors, while soluble immunostimulants are rapidly drained from tumors. Liu *et al.* demonstrated that CpG can be retained locally in tumors by conjugating CpG to lipophilic moieties, which readily insert into tumor cell membranes after *i.t.* injection [140]. A strategy reported by Intra *et al.* involves activation of the immune system to clear tumor cells after resection to prevent relapse. In this study, PLGA sutures closing the incision from resection were used to encapsulate and sustain the release of CpG locally at this site [38]. Using these CpG-loaded sutures during resection of N2a neuroblastoma–an extracranial pediatric cancer–protected mice from relapse and prolonged survival compared to mice sutured with unloaded PLGA and locally injected with CpG.

**Figure 5:**
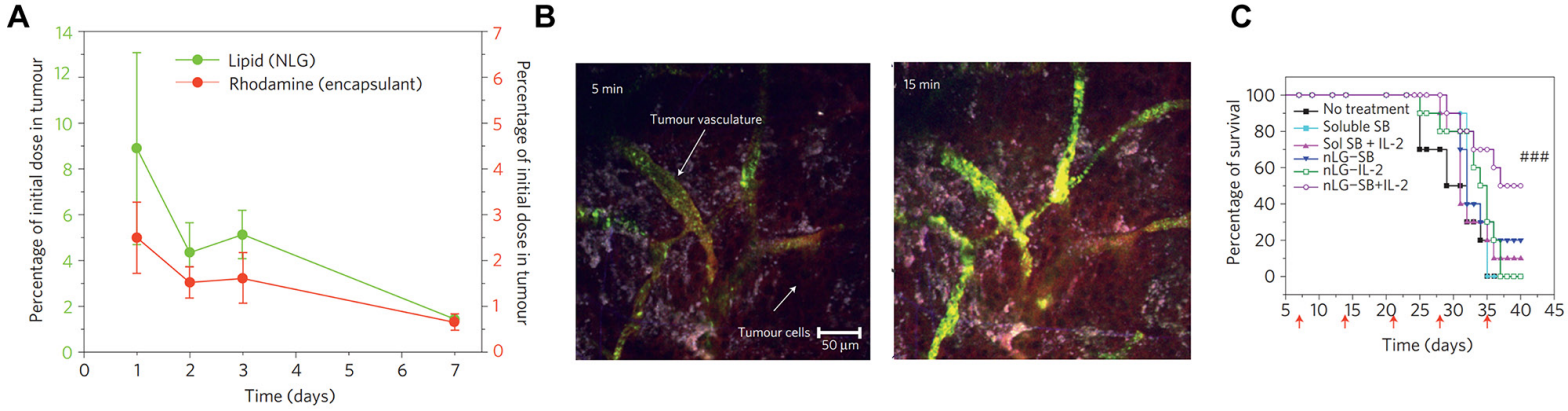
Systemically-administered nLGs accumulate in tumor vasculature and enhance anti-tumor immunity **A.** nLG and encapsulated cargo localize to and are retained in tumors over time after intravenous injection. **B.** Imaging of nLGs (green) in tumor vasculature. **C.** Therapeutic treatment with nLGs encapsulating IL-2 and TGF-β1 inhibitor enhance survival compared with soluble components or IL-2/TGF-β1 delivered alone in nLG. Adapted with permission from Macmillan Publishers Ltd: Nature Materials [143] copyright (2012).

In addition to targeting the tumor microenvironment with immunostimulants, strategies aimed at neutralizing immunosuppressive cytokines may also allow anti-tumor immunity to overcome the suppressive mechanisms of the tumor microenvironment. IL-2 therapy, for example, shows potential by augmenting the activation and expansion of anti-tumor CTLs. Clinical trials testing IL-2 during metastatic melanoma demonstrate 4-6% of patients achieve complete remission [141, 142]. One major barrier limiting the robustness of this idea is the restraint of IL-2 induced expansion of CTLs by TGF-β and other immunosuppressive cytokines present in tumors [115]. Park *et al.* has enhanced IL-2 based immunotherapy in mice using nanoparticles co-loaded with IL-2 and a small molecule inhibitor of TGF-β1 (SB505124) [143]. In this strategy nanoparticle liposomal polymeric gels (nLGs) were designed. The nLGs were formed from a biodegradable polymer core loaded with drug solubilized in cyclodextrans and then enveloped in a PEGylated lipid shell. This design allowed efficient encapsulation and co-delivery of both hydrophilic IL-2 and hydrophobic SB505124. Passive targeting due to the EPR effect led to nLG accumulation in both subcutaneous tumors (Figure 5A, 5B) and metastatic pulmonary tumors after nLGs were administered intravenously to mice bearing B16-F10 tumors. Weekly administration of nLGs encapsulating IL-2 and SB505124 protected mice from tumors and enhanced survival compared to soluble systemic treatment, which conferred no benefit compared with untreated mice (Figure 5C). Mechanistically, these therapeutic effects correlated with increased NK cell frequencies in tumors and improved CTL recruitment. These examples demonstrate new ways in which biomaterial strategies might help overcome inactivation of CTLs in the tumor microenvironment, a challenge that connects with the hurdle discussed next, heterogeneity of disease.

### Addressing heterogeneity of disease

A major barrier to cancer immunotherapy is the highly heterogeneous nature of tumors within individual patients, in different types of cancer, and across patient populations [3, 144, 145]. Tumor cells are dynamic, exhibiting phenotypic and antigenic heterogeneity that creates variable susceptibleness to different vaccines and immunotherapies, as well as evolution of these responses as treatment continues or during relapse [144].

The effectiveness of a cancer vaccine depend in part on the individual MHC alleles expressed by the patient and the level of expression of TAAs targeted by the vaccine [146]. Frequent mutations or immunoselection in response to vaccination may drive the loss of expression of targeted antigens [147]. One potential strategy to overcome this challenges is development of personalized vaccines for individual patients. For example, antigens present in a patient's tumor that exhibit a high affinity for their individual MHC alleles could be identified and utilized in antigen selection [148]. Additionally, vaccines might utilize multiple patient specific TAAs for a broad immune response less susceptible to loss of antigens or antigenic editing by tumors [149]. However, while vaccines in the clinic targeting well characterized TAAs, such as in melanoma, have been effective at causing partial tumor regression, complete regression is rare, likely due to antigenic shift of tumors in response to immune attack of tumors induced by vaccination [147, 150]. Importantly, recent preclinical studies reveal neoantigens driven by mutations are seen as non-self and preferentially targeted by CTLs after checkpoint blockade [151]. This outcome suggests neoantigens–if identifiable during treatment–could be used effectively for vaccination by pairing with checkpoint bloackade [152, 153]. A majority of neoantigens driven by mutation are patient-specific, and one current challenge is that the genome of a patient's own tumor cells must be sequenced for identification of the neoantigens if prepared *de novo*.

Resected tumors theoretically contain all antigens present in an individual's tumor, including neoantigens resulting from mutations. Thus these tissues may be used as effective antigen sources in personalized cancer vaccines. Using irradiated tumor cells or tumor lysates as antigen sources in vaccines may likewise offer the potential to better combat tumor immunoselection [149]. Biomaterials can further enhance this approach by targeting to immune cells, sustained delivery of antigens, and co-localization with adjuvants–all discussed previously for cancer vaccines based on well-defined antigens. The previously-discussed scaffold vaccines from the Mooney lab employed irradiated whole tumor cells or tumor lysates as a source of antigens. PLGA MP and NP loaded with tumor lysates have also been developed [154] and have recently been shown to enhance immunogenicity in preclinical breast cancer models [154, 155]. Another interesting idea recently reported is the use of exosomes secreted from DCs after DCs were stimulated with PolyIC and loaded with tumor lysates to create an effective vaccine in murine models of melanoma [156].

Advances in sequencing technologies have enabled efficient exosome mapping of tumor cells [157]. In this area, biomaterials and nanotechnology are rapidly developing as powerful tools to enable capture of rare circulating tumor cells (CTCs) [11, 158–162], capabilities that may enhance personalized cancer immunotherapy by allowing identification of neoantigens in individual patients [148]. This approach might open new potential even for patients with haematological tumors or unresectable solid tumors. Additionally, capture of CTCs may allow for early detection of cancer and patient-specific drug or immunotherapy screening to determine the best treatment options [65].

**Figure 6:**
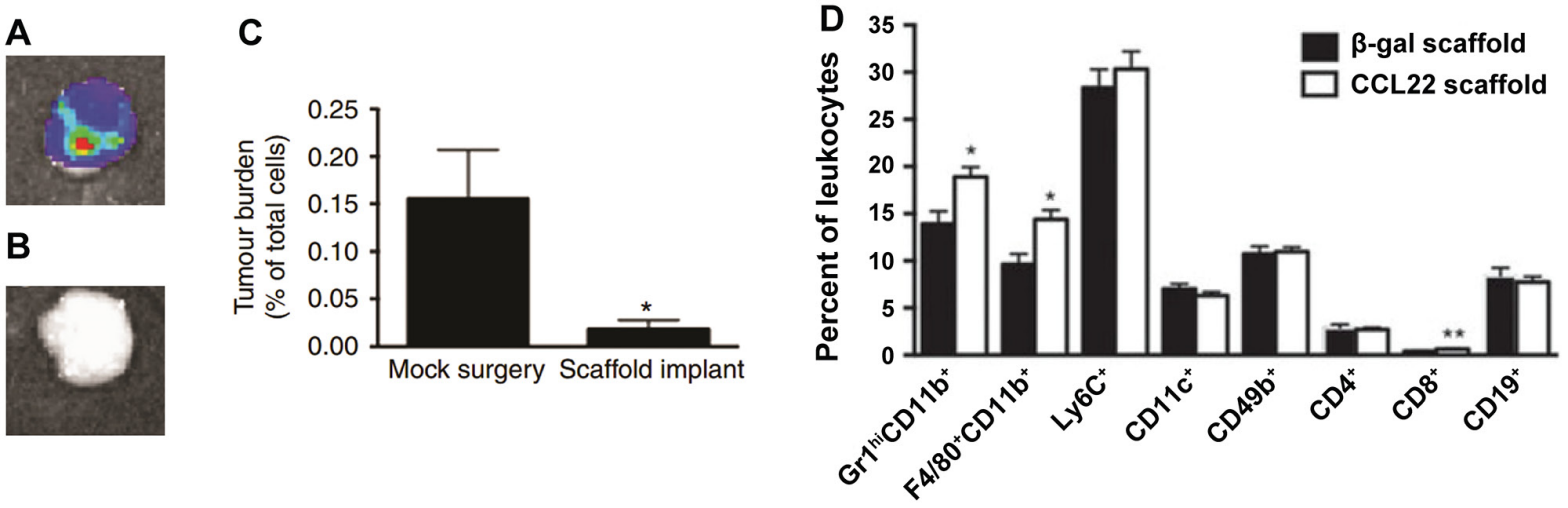
Capture of metastatic tumor cells through implantable scaffolds mimicking the pre-metastatic niche Metastatic tumor cells accumulate in scaffolds implanted in **A.** the peritoneal fat-pad, but **B.** not in the fat-pad of scaffold-free mice. **C.** Metastatic tumor cells migrating to scaffolds result in lower metastatic tumor burden in lungs. **D.** Scaffolds delivering a CCL22 vector increase the accumulation of Gr1^hi^CD11b^+^ cells compared with a control vector (β-galactosidase). Adapted by permission from Macmillan Publishers Ltd: Nature Communications [137] copyright (2015).

Another new strategy aimed at CTCs is based on altering or exploiting the homing of these cells. Azarin *et al.* recently demonstrated a biomaterial strategy that enables efficient capture of CTCs using implantable scaffolds, which function to preferentially redirect metastatic tumor cells from other solid organs [137]. In this study PLGA scaffolds were designed to mimic the pre-metastatic niche–an environment consisting of matrix proteins, cytokines and chemokines as well as immune cells–to create a conducive location for new tumors from circulating metastatic cells. Tumor cells accumulated in scaffolds after subcutaneous or intra-peritoneal implantation of scaffolds in mice bearing breast cancer tumors in the mammary fat pads (Figure 6A) [137]. In contrast, tumor cells were not detected in the peritoneal fat pads of mice not implanted with scaffolds (Figure 6B). Additionally, the accumulation of metastatic tumor cells in scaffolds correlated with decreased incidence of metastases in the lungs and liver, as well as decreased metastatic tumor burdens in the lungs (Figure 6C). Mechanistically, the recruitment of CTCs was mediated by the infiltration of leukocytes (Figure 6D). These infiltrating leukocytes consisted of populations of immune cells at similar relative levels to those seen in the lungs of tumor bearing mice, which are sites of frequent metastasis. In particular Gr1^hi^CD11b^+^ MDSCs accumulated in scaffolds to a high degree, consisting of 66% of total leukocytes in the scaffold, compared to 89% of total leukocytes in the lungs 28 days after tumor inoculation [137]. By utilizing scaffolds to locally deliver lentiviral vectors encoding the chemokine CCL22, Gr1^hi^CD11b^+^ cell infiltration in the scaffold was increased significantly by day 14 after implantation, compared to scaffolds delivering a control vector (Figure 6D). Additionally, this chemokine increased infiltrating Gr1^hi^CD11b^+^ cells to enhance tumor cell capture in the scaffold. This study also demonstrated a potential strategy to address heterogeneity of disease with respect to metastatic potential, where early metastases can be detected by label-free *in situ* imaging of scaffolds based on optical properties of cancer cells. This capability may allow for patients to be treated based on expected aggressiveness of their disease. Additionally, metastatic cancer cells may be removed from scaffolds or other “sensor” devices for analysis of biomarkers or antigen expression. An especially exciting aspect is the multi-functionality that these types of devices could play as both a diagnostic tool to address heterogeneity, and as a strategy to delay or inhibit metastases to allow time for parallel treatments to work.

## CONCLUSIONS

It is clear that immunotherapies for cancer treatment have progressed greatly in recent years, revealing new possibilities through which the immune system can be used to treat cancer. The successes and failures of immunotherapies in the clinic have given researchers newfound understanding of the significant challenges presented by cancers that limit current immunotherapies. Nanotechnologies have been investigated extensively for cancer immunotherapies and vaccination pre-clinically, but have had comparatively little impact for patients thus far. One common occurrence is clinicians and engineers coupling cutting-edge ideas from their respective field with basic–or sometimes outdated–ideas from the other field. For example, much nanoparticle research is being performed on small molecules drugs delivered as a monotherapy that are never used as a primary therapy in patients. Similarly, most clinical therapies involve systemic delivery, while many pre-clinical engineering strategies exist for targeting specific cells or tissues. Further, as can be observed by range of papers reviewed here, a great deal of pre-clinical work involving biomaterials is conducted in fairly basic models–for example, B16-F10. Although these are valuable starting points, utilization of more sophisticated animal models that recapitulate cardinal features–such as pathology and metastatic tendency–could improve the translatability of new material strategies. This gap highlights the potential for impact that materials can have as the focus on cross-disciplinary understanding and collaboration grows; there is outstanding synergy at the interface of nanotechnology, bioengineering, and immunotherapy. This nexus creates enormous opportunities for teams of researchers that understand both the clinical and technological needs of new cancer therapies.
